# Snake words in Estonia: Language, nature and extinction in Andrus Kivirähk’s The Man Who Spoke Snakish – ERRATUM

**DOI:** 10.1017/ext.2024.11

**Published:** 2024-05-15

**Authors:** Alfie Howard, Diane Nelson

Cambridge University Press and Assessment would like to issue an apologise. The affiliation of Diane Nelson was originally given as School of English, University of Leeds. The correct affiliation for Diane Nelson is School of Languages, Cultures and Societies, University of Leeds. The article has been corrected.
